# Aerosolized SARS-CoV-2 transmission risk: Surgical or N95 masks?

**DOI:** 10.1017/ice.2020.465

**Published:** 2020-09-15

**Authors:** Petrick Periyasamy, B. H. Ng, Umi K. Ali, Zetti Z. Rashid, Najma Kori

**Affiliations:** 1 Department of Medicine, Universiti Kebangsaan Malaysia Medical Centre, Kuala Lumpur, Malaysia; 2 Department of Medical Microbiology and Immunology, Universiti Kebangsaan Malaysia Medical Centre, Kuala Lumpur, Malaysia

*To the Editor—*Based on available evidence, coronavirus disease 2019 (COVID-19) is thought to spread through close contact and droplet transmission. However, some have debated that it could be airborne. Airborne transmission occurs when particles of <0.5 μm within droplets spread through exhaled air via a process called aerosolization. These particles can remain in the air for long periods and can disseminate over distances >1 m. In the context of COVID-19, airborne particles can occur during certain aerosol-generating procedures (AGPs). The World Health Organization (WHO) underlines the use of N95 respirators or equivalent as part of personal protective equipment (PPE) for healthcare workers (HCWs) managing COVID-19–positive patients when AGPs are being conducted.

This retrospective observational study describes the result of reverse-transcriptase polymerase chain reaction (RT-PCR) testing for severe acute respiratory coronavirus virus 2 (SARS-CoV-2) in HCWs wearing different form of PPE who had close contact with a confirmed COVID-19 patient during performing AGPs. All HCWs were quarantined for 14 days after the exposure. SARS-CoV-2 RT-PCR nasopharyngeal swabs were performed at different intervals. Little is known about the effectiveness of different types of PPE for preventing COVID-19 in HCWs. We describe the clinical outcome of HCWs exposed to sudden acute respiratory infection patient before the diagnosis of COVID-19 was known.

## Case report

A 70-year-old man with known ischemic heart disease and dyslipidemia presented with severe headaches and cough for 1 week. He had a recent history of travelling to Russia in December 2019 and Jakarta in early February 2020. On presentation, no screening for SARS-CoV-2 was performed as Russia and Jakarta have not been flagged as epidemiological links to COVID-19 by the Malaysian Ministry of Health.

In the emergency department, he was tachypneic with respiratory rate of 28 breaths per minute, oxygen saturation of 86% in room air and requiring oxygen supplement of 40% via venturi mask. His condition worsened, requiring noninvasive mechanical ventilation (NIV); NIV failed and he was intubated. While awaiting transfer to the intensive care unit, manual ventilation via bag–valve–mask was performed. His chest radiography showed bilateral ground-glass opacities, mainly in the lower lobes.

In view of sudden acute respiratory infection, nasopharyngeal (NP) swabs were sent for SARS-CoV-2 real-time reverse-transcriptase polymerase chain reaction (RT-PCR) testing. Overall, 25 HCWs were exposed to AGPs by this severe pneumonia patient who later tested positive for SARS-CoV-2. These procedures included nebulizer therapy, endotracheal intubation, invasive ventilation, and tracheal suctioning. The mean time of exposure was 34.4 minutes (range, 15–180 minutes). All 25 at-risk HCWs were placed on home quarantine for 14 days. They were monitored for cough, sore throat, headaches, myalgia and dyspnea. All HCWs with different levels of PPE and exposure times finally tested negative for SARS-CoV-2.

**Table 1.** Types of the AGPs With the Type of PPE Used and Timing of COVID RT-PCR From Initial Exposure

		No. of HCWs and Timing of COVID-19 RT-PCR NP Swab From Exposure			
Type of AGP (n = 25)	PPE	Day 1	Day 9	Day 13	Day 15
Exposure to aerosol from VM, nebulizer, or oral suctioning (n = 15)	Surgical mask	15	15	13	15
	N95 mask	0	0	0	0
Intubation (n = 3)	Surgical mask	0	0	0	0
	N95 mask	3	0	3	0
Ventilation (n = 7)	Surgical mask	4	4	2	4
	N95 mask	3	0	3	0

Note. AGP, aerosol-generating procedure; PPE, personal protective equipment; VM, ventimask; NP, nasopharyhgeal swab.

No. of HCWs and day of repeat NPO swab as above. All tested negative at each sampling.

## Discussion

COVID-19 is a very contagious disease that poses an occupational health risk to HCWs. SARS-CoV-2 transmission is believed to occur mainly through respiratory droplets. Current guidelines recommend the use of N95 masks and goggles during AGPs when attending to COVID-19 patients because the virus may become airborne under certain conditions. Respiratory PPE is particularly important to reduce the risk of respiratory infection in HCWs. A variety of PPE that provides different degrees of respiratory protection: medical face masks, respiratory protection equipment, goggles, and face shield. The size of the virus particle, the distance it can travel, and how deeply the virus can penetrate the host’s respiratory tract are determinants of required PPE.

Medical masks have a fluid-resistant outer layer designed to prevent a stream of liquid entering the mouth. Medical masks are able to filter large particles but are not certified to protect users from airborne infections. Data concerning how well medical masks work against SARS-CoV-2 are lacking. The N95 is a type of respirator able to filter out both large and small airborne particles. Factors that may affect the efficacy of N95 masks includes whether the HCW is trained in wearing N95 respirator and whether a fit-test was conducted. In one study comparing fit-testing with no fit-testing, there was no difference in respiratory infection risk between the 2 groups.^1^

The previous study claimed that there was insufficient evidence regarding the superiority of N95 masks over medical masks in protecting HCWs from transmissible acute respiratory infections in clinical settings.^2^ A study from Singapore reported on 41 HCWs exposed to an unknown COVID-19 patient during an AGP for >10 minutes, of whom 85% wore only surgical masks. All tested negative for SARS-CoV-2 by RT-PCR.^3^

A systematic review of 4 randomized controlled trials on masks showed that medical masks and N95 respirators offer similar protection against viral respiratory infection, including coronavirus, for HCWs during non–AGPs.^4^ The effectiveness of medical masks in protecting HCWs from SARS was inconsistent, and differing levels of exposure may explain such discrepancies. Xiao et al^5^ reported that masks did not prevent the transmission of influenza in 7 studies. On the contrary, Jefferson et al^6^ suggested that wearing masks significantly reduced the risk of SARS transmission.

Laboratory experiments have shown that SARS-COV-2 may remain viable for up to 3 hours, but clinical data have not demonstrated conclusively that SARS-CoV-2 is frequently spread via long distance airborne nuclei during routine care or following AGPs.^7^ All HCWs with different levels of PPE and exposure time tested negative for SARS-CoV-2. These findings are consistent with the meta-analysis, which showed the use of both N95 respirators and medical masks was associated with up to 80% reduction in risk of SARS.^8^ Other than the PPE that wore by our HCWs, we believe that the rate of clearance of aerosols may also affect the risk of infection in HCWs. Our general wards have around ~6 air exchanges per hour, which reduced air contaminants, assuming that a single air exchange eliminates 63% of airborne contaminants.^9^

In the case we presented, none of our HCWs wore N95 masks nor goggles. However, none of the 25 individuals at risk developed major symptoms, and serial NP swabs have proven that not one of them acquired the infection (Table 1). Our observation is therefore consistent with previous reports that have been unable to show that N95 masks were superior to 3-ply masks in preventing transmission to HCWs performing AGPs. Further randomized control trial on ascertaining the effectiveness of the N95 respirators or medical masks in preventing HCWs from SARS-CoV-2 are warranted.
